# A Deadly Duet: Fanconi Anemia (FA) With Head and Neck Cancer

**DOI:** 10.7759/cureus.95448

**Published:** 2025-10-26

**Authors:** Chaithanya D, Lakshmi JS, Christopher John, Satish Srinivas K

**Affiliations:** 1 Radiation Oncology, Sri Ramachandra Institute of Higher Education and Research, Chennai, IND

**Keywords:** chemotherapy-induced myelosuppression, conventional radiotherapy, fanconi anemia, head and neck cancer, inherited disorder

## Abstract

Fanconi anemia (FA) is a rare, inherited disorder characterized by chromosomal instability, bone marrow failure, and predisposition to malignancies, including head and neck squamous cell carcinoma (HNSCC). The treatment of HNSCC in FA is challenging due to the extreme sensitivity of these patients to DNA-damaging agents. A 39-year-old male with FA presented with odynophagia and neck swelling. Examination revealed a mass at the base of the tongue (BOT) with bilateral cervical lymphadenopathy. Histopathology confirmed moderately differentiated squamous cell carcinoma (MD-SCC) (p16 negative). He underwent induction chemotherapy but developed severe myelosuppression. In view of the unusual clinical presentation, a mitomycin stress test was performed, confirming FA. Consequently, further chemotherapy was deferred, and radiotherapy was delivered with careful monitoring and supportive care. The patient achieved a complete metabolic response post-treatment. The case highlights the importance of radiation treatment in FA patients with exaggerated chemotherapy toxicities and the need for tailored management in this patient population.

## Introduction

Fanconi anemia (FA) is a rare autosomal recessive disorder with an estimated incidence of one in 136,000 live births, although estimates range between one in 100,000 to 250,000 [1]. FA is extremely uncommon among Asian populations and more prevalent among South African Afrikaner and Ashkenazi Jew populations [1]. FA patients are 500 times more likely than the general population to acquire solid and hematological malignancies such as head and neck squamous cell carcinoma (HNSCC) and leukemia [2,3]. By the age of 40 years, cumulative HNSCC incidence reaches 14% [3]. HNSCC in FA typically presents at a younger age (20-50 years) and is often diagnosed at advanced stages [1].

The risk factors for HNSCC in FA are not fully defined but include defective DNA repair, age, and graft-versus-host disease following hematopoietic cell transplantation, all of which play a significant role [1]. Mutations in any of the 22 genes involved in the DNA repair pathway result in FA. Defective DNA interstrand crosslink repair leads to genomic instability, bone marrow failure, and cancer predisposition [1]. Standard cancer treatment is complex because patients are hypersensitive to DNA-damaging chemicals, such as chemotherapy and radiation [1].

## Case presentation

**Figure 1:**
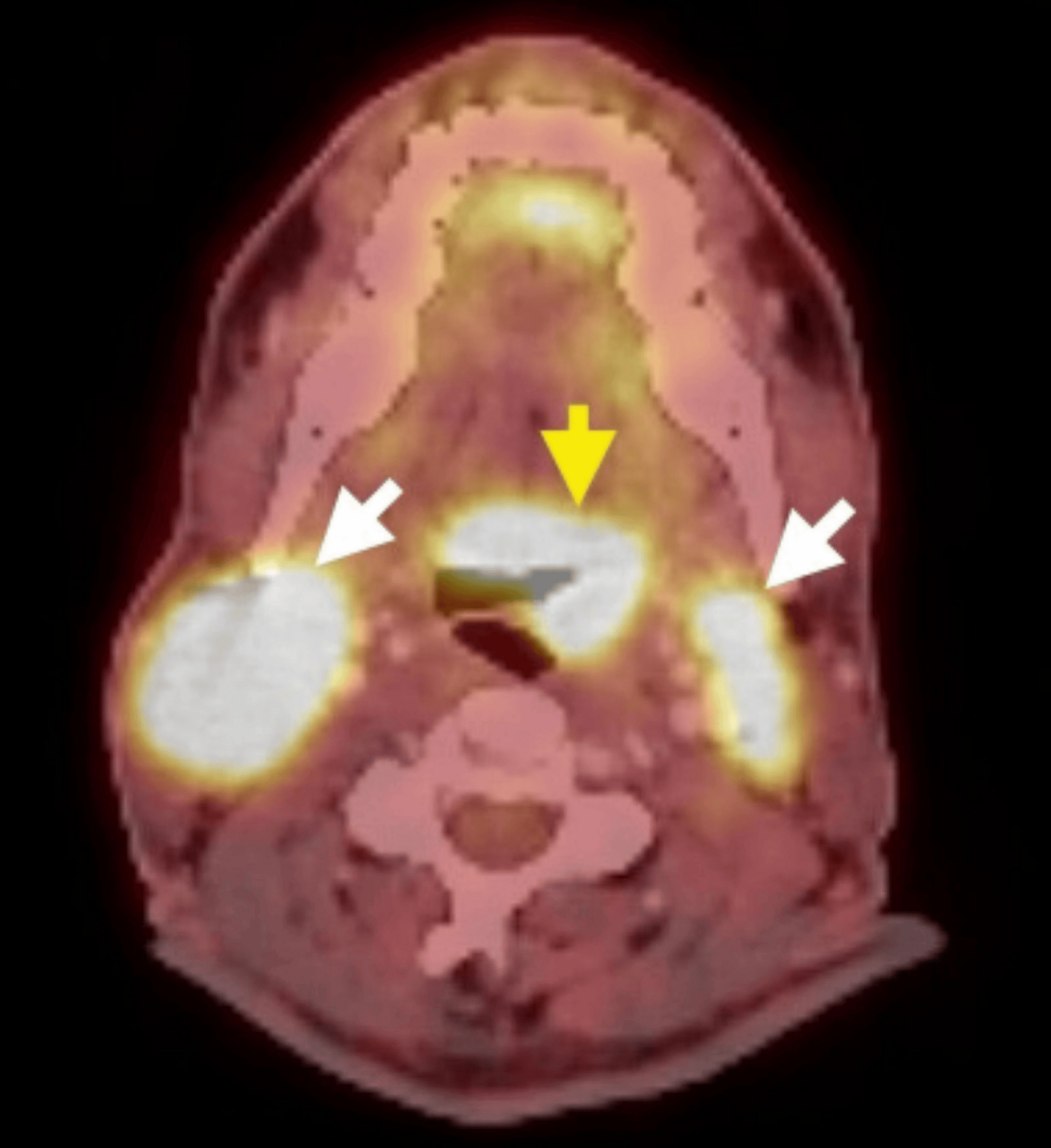
Axial PET fusion done prior to treatment. The imaging shows a fluorodeoxyglucose (FDG)-avid, enhancing lesion involving the base of the tongue, vallecula, and epiglottis on the left (yellow arrow), as well as enlarged bilateral level 2 cervical lymph nodes (white arrows).

**Figure 2:**
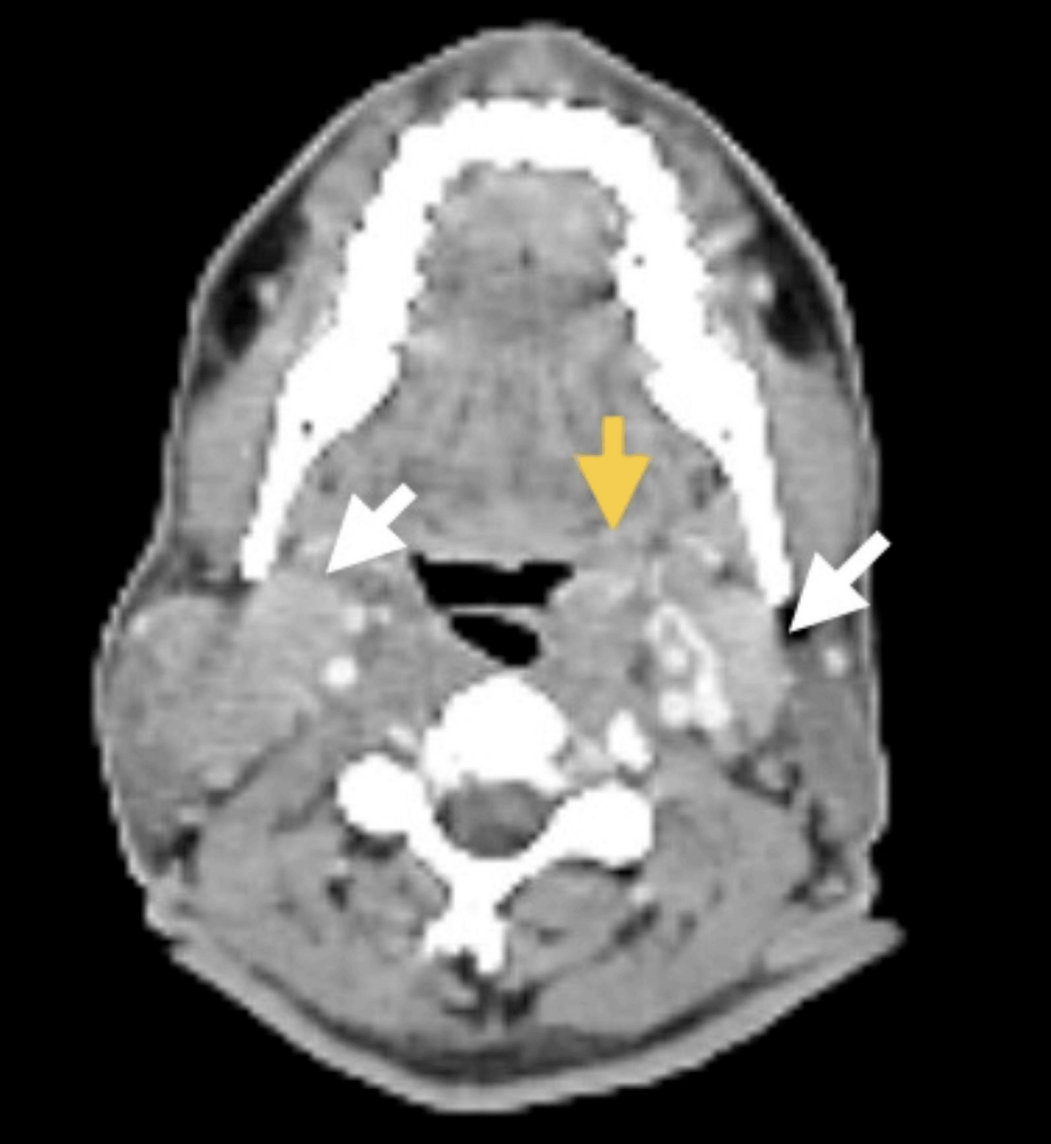
Axial contrast CT done prior to treatment. The imaging shows a fluorodeoxyglucose (FDG)-avid, enhancing lesion involving the base of the tongue, vallecula, and epiglottis on the left (yellow arrow), as well as CT-enhancing, enlarged bilateral level 2 cervical lymph nodes (white arrows).

A 39-year-old male of short stature with low-set ears, a non-smoker, and a history of young-onset type 2 diabetes mellitus presented with odynophagia and neck swelling (right) over two months since December 2022. Clinical examination revealed a growth at the base of the tongue (BOT) extending to the pharyngoepiglottic fold, with bilateral cervical lymphadenopathy. A moderately differentiated squamous cell carcinoma (MD-SCC) that was p16-negative was discovered during a biopsy. PET-CT imaging revealed bilateral fluorodeoxyglucose (FDG)-avid cervical lymphadenopathy with extranodal extension (clinically staged as cT3N3bM0) and an FDG-avid lesion including the glossotonsillar sulcus, vallecula, and base of tongue (Figures 1, 2).

**Figure 3:**
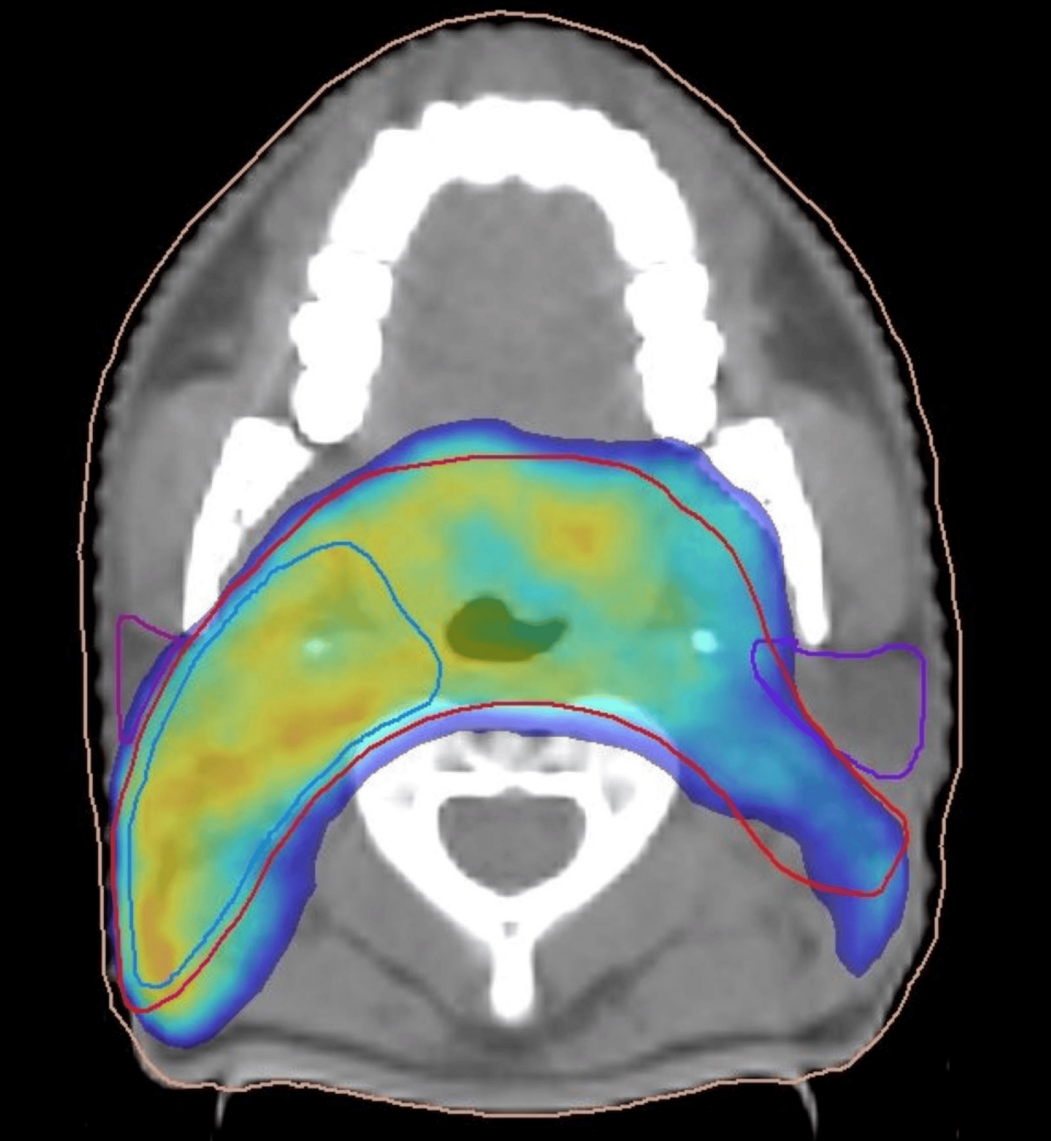
Axial CT slice of a head and neck intensity modulated radiotherapy (IMRT) plan. The dose distribution shows conformal coverage of the primary tumor and nodal planning target volume (PTVs) with sparing adjacent organs at risk.

**Figure 4:**
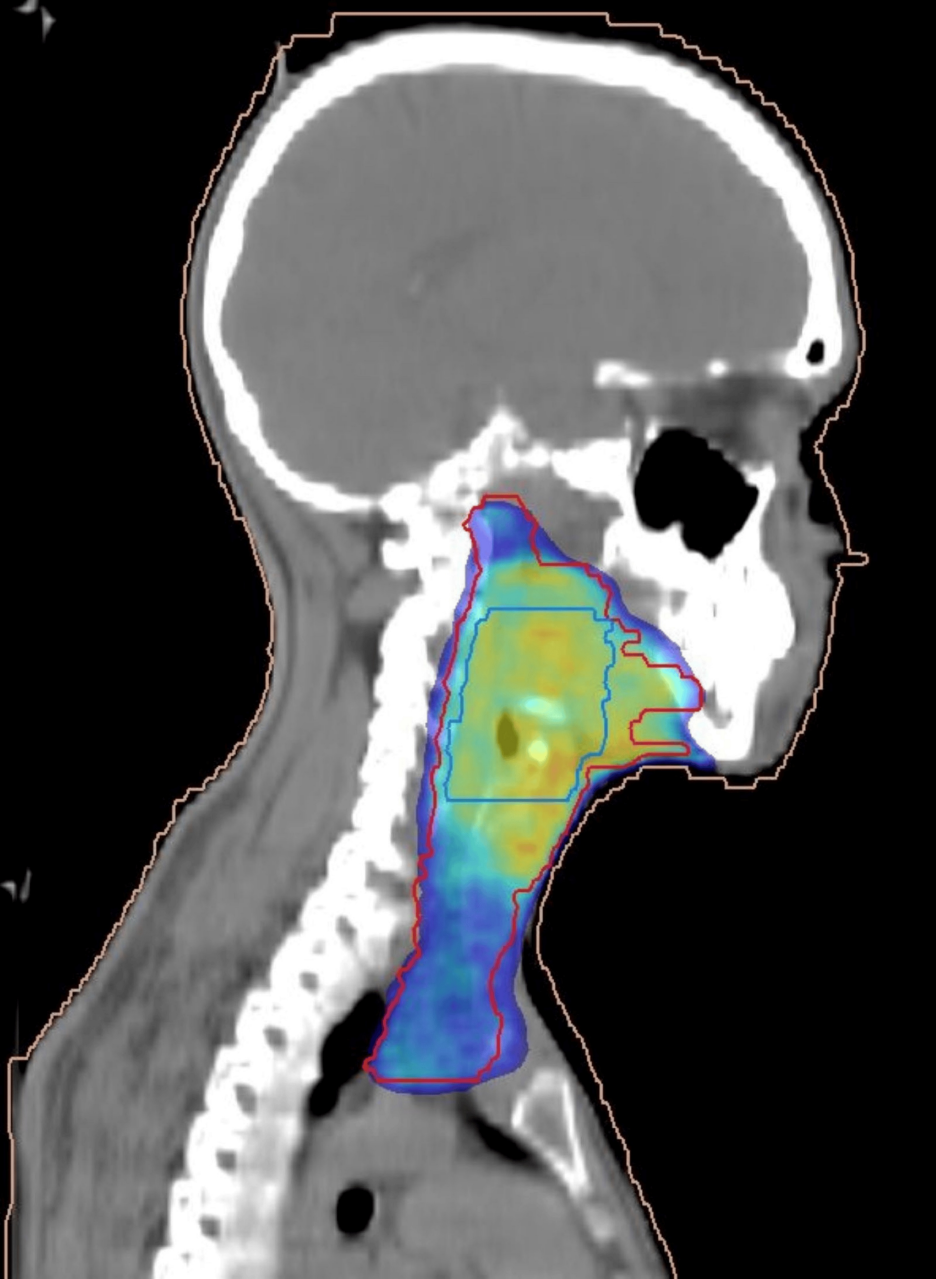
Sagittal CT slice of the same plan. The isodose wash demonstrates adequate target coverage with sharp dose fall-off to protect the spinal cord and surrounding normal structures.

The case was discussed in a multidisciplinary tumor board meeting, and it was planned for induction chemotherapy followed by concurrent chemoradiotherapy (CCRT). However, one week following the first cycle of chemotherapy with cisplatin (100 mg) and docetaxel (100 mg), the patient developed grade 3 myelosuppression requiring hospital admission and appropriate supportive measures. The patient recovered from hematological toxicity and received dose-reduced chemotherapy with carboplatin (300 mg) and docetaxel (80 mg), but subsequently developed grade 4 myelosuppression. Because of unexplained severe myelosuppression and significant clinical appearance, a mitomycin stress test was performed, which revealed a considerable increase in chromatid exchange and radial formations, confirming FA. Considering severe myelosuppression, further chemotherapy was deferred and proceeded with radiation therapy (RT). He received radiotherapy, a total dose of 6600 cGy in 33 fractions, five days a week over 6.5 weeks using the intensity modulated radiotherapy (IMRT) technique (Figures 3, 4).

**Figure 5:**
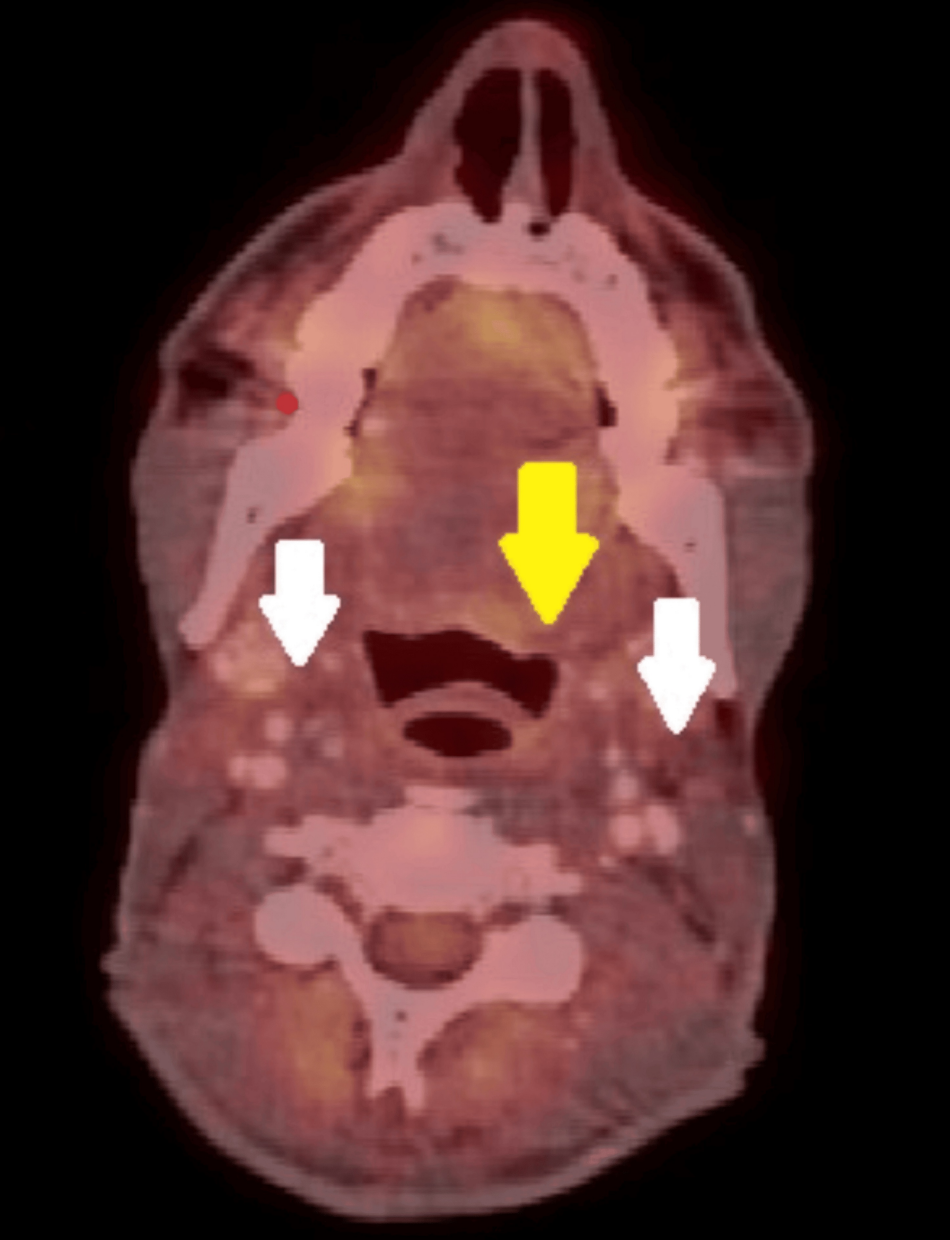
Axial FDG PET-CT fusion of the head and neck region done post-treatment. The imaging shows complete resolution with no fluorodeoxyglucose (FDG) uptake of the primary lesion (yellow arrow) and bilateral cervical lymph node region (white arrows), suggestive of complete treatment response.

**Figure 6:**
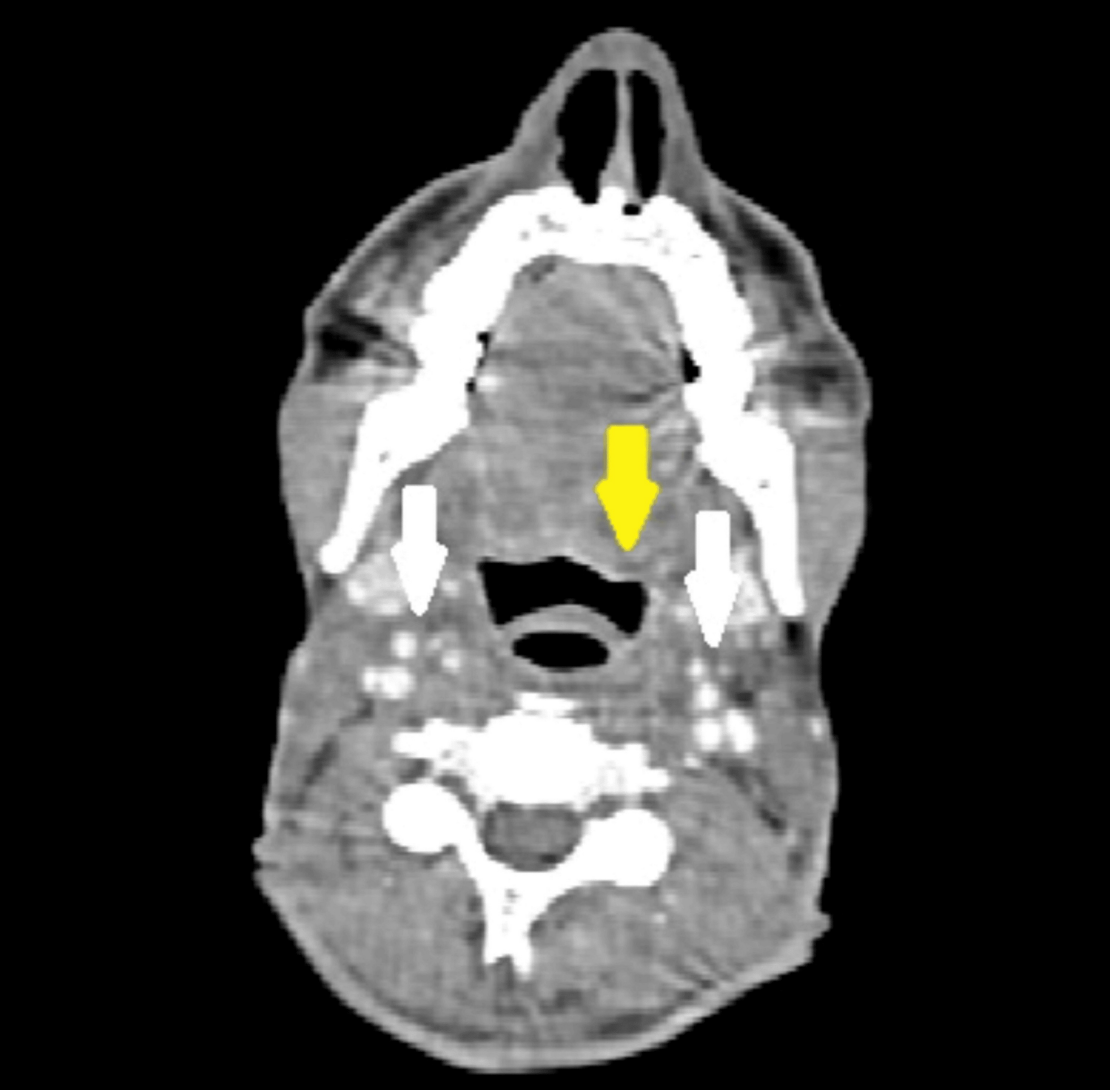
Axial contrast-enhanced CT of the head and neck region. There is no obvious mass or thickening seen in the primary region (yellow arrow) and no bilateral cervical lymphadenopathy (white arrows).

During radiotherapy, the patient developed grade 3 dermatitis, grade 3 mucositis, and odynophagia. He was managed with supportive care, including nasogastric tube placement, granulocyte-colony stimulating factor (G-CSF), and antibiotics as required. He was continuously monitored with a biweekly complete blood count during RT treatment. On completion of radiotherapy, response assessment imaging at eight weeks showed a complete metabolic response (Figures 5, 6) (Table 1).

**Table 1:** Serial hematological parameters during treatment.

Details	Hemoglobin (g/dL)	Total Leucocyte Count (cells/cumm)	Polymorphs (%)	Absolute Neutrophil Count (cells/µL)	Platelet Count /mm^3^	Packed Cell Volume (%)
Reference range	12-17 g/dL	4,000 – 11,000/mm^3^	45-70%	More than 1500 cells/ µL	150,000 -450,000/mm^3^	36-50%
Prior to chemotherapy	15.3 g/dL	6,620/mm^3^	52.8%	3,495 cells/ µL	304,000/mm^3^	48.1%
Post 1st induction chemotherapy	12.2 g/dL	410/ mm^3^	0	0	74,000/mm^3^	37.5%
Post 2nd induction chemotherapy	8.3 g/dL	360/ mm^3^	3%	72 cells/ µL	53,000/mm^3^	25.3%
Post radiation therapy	11.7 g/dL	5,570/ mm^3^	87%	4,846 cells/ µL	187,000/mm^3^	34.8%

Thus, this patient has remained under follow-up to date, with clinical and radiological assessments, and has reported being disease-free for over two years.

## Discussion

FA is a hereditary genetic disorder that typically manifests in the second to fourth decade of life and is characterized by severe pancytopenia leading to bone marrow failure [1]. Mutations in any of the 22 FA/BRCA DNA repair pathway genes cause chromosomal instability by failing to repair DNA interstrand cross-links [1]. This underlying defect affects both embryonic development and postnatal tissue homeostasis, leading to a complex, multisystem syndrome marked by congenital malformations and progressive bone marrow failure, as well as a markedly increased risk of cancer [1]. Congenital anomalies are present in approximately 60-75% of FA patients and often provide the earliest diagnostic clues. Skeletal anomalies such as absent or hypoplastic thumbs and radii in the upper limb are classic features. Other anomalies may include short stature, microcephaly, microphthalmia, skin hyperpigmentation, and renal malformations such as horseshoe or absent kidneys [1]. These defects arise from impaired DNA repair during embryogenesis, leading to increased apoptosis and abnormal tissue development, particularly in mesoderm-derived structures.

However, the phenotypic spectrum of FA is highly variable, and some patients may lack overt anomalies, underscoring the need for clinical vigilance in children presenting with growth retardation, dysmorphic features, or unexplained cytopenias [1]. FA patients have a 500 times increased risk of head and neck and anogenital squamous cell carcinomas (SCC) in adolescents and young adults, and 800 times the risk of acute myeloid leukemia (AML) [2,3].

The diagnosis of FA relies on cytogenetic testing, with the chromosome breakage test using a clastogenic agent that involves "mitomycin C (MMC)" or "diepoxybutane (DEB)" remaining the gold standard [1]. FA cells demonstrate markedly increased chromosomal breakage and characteristic radial figures when exposed to these agents [1]. In this case, the patient's severe hematological toxicities following standard chemotherapy prompted evaluation for FA, which was confirmed by a positive MMC stress test showing a significantly higher percentage of cells with radial figures compared to standard controls [1]. This finding reinforces the utility of the MMC test, especially in resource-limited settings where molecular testing may not be readily available [1]. However, it is essential to acknowledge its limitations: patients with somatic mosaicism or hypomorphic mutations may exhibit normal or borderline results, necessitating a comprehensive diagnostic approach that incorporates clinical, cytogenetic, and molecular findings [1].

Management of FA requires a multidisciplinary approach. Early diagnosis is crucial for optimizing outcomes, as timely referral for hematopoietic stem cell transplantation (HSCT) can be life-saving in bone marrow failure [1]. Cancer surveillance is essential given the high risk of hematological and solid malignancies [1]. Prophylactic strategies, including human papillomavirus (HPV) vaccination, lifestyle modifications (such as smoking and alcohol avoidance), and regular screening, are key components of long-term care [1]. Genetic counseling is critical for guiding family planning and enabling cascade testing of at-risk relatives [1].

Because of the underlying DNA repair deficiency, which makes patients extremely vulnerable to DNA-damaging treatments like chemotherapy and radiation, treating HNSCC in FA is complicated [1]. Surgery is generally preferred for oral cavity tumors, whereas radiotherapy remains the mainstay for oropharyngeal, laryngeal, and hypopharyngeal cancers, albeit with careful dose modification and close monitoring for toxicity [4]. Despite high rates of acute toxicity and treatment breaks, some patients achieve good tumor responses [4]. Recurrence, second primary tumors, and a five-year survival rate of less than 50% remain risks [4].

For patients with HNSCC, the occurrence of unusually high treatment-related toxicity should raise the possibility of an underlying FA [2,3]. Such cases should be managed through a multidisciplinary approach, which balances disease control with minimizing the adverse effects carefully [1,4]. Close clinical observation, proactive supportive care, and proper modification of radiotherapy and chemotherapy doses are essential in ensuring maintenance of safety in addition to therapeutic benefit [4]. Moreover, severe lifestyle interventions, especially total abstinence from tobacco and alcohol and examination of the oral cavity at regular intervals, are important in decreasing risk and enhancing the outcome in the susceptible population [1,3].

## Conclusions

FA is a complex, multisystem disorder that demands a high index of suspicion, particularly in children with congenital anomalies, growth failure, or cytopenia. The chromosomal breakage test remains a valuable diagnostic technique, but its interpretation should be considered in conjunction with genetic and clinical data. For patients with this uncommon and difficult ailment, improved outcomes require early diagnosis, interdisciplinary care, and ongoing monitoring.
